# The feasibility of a noise elimination method using continuous wave response of therapeutic ultrasound signals for ultrasonic monitoring of high-intensity focused ultrasound treatment

**DOI:** 10.1007/s10396-021-01083-5

**Published:** 2021-04-01

**Authors:** Ryo Takagi, Toshikatsu Washio, Yoshihiko Koseki

**Affiliations:** Health and Medical Research Institute, National Institute of Advanced Industrial Science and Technology (AIST), Tsukuba, Japan

**Keywords:** High-intensity ultrasound, Ultrasound imaging, Image processing, Noise elimination

## Abstract

**Purpose:**

In this study, the robustness and feasibility of a noise elimination method using continuous wave response of therapeutic ultrasound signals were investigated when tissue samples were moved to simulate the respiration-induced movements of the different organs during actual high-intensity focused ultrasound (HIFU) treatment. In addition to that, the failure conditions of the proposed algorithm were also investigated.

**Methods:**

The proposed method was applied to cases where tissue samples were moved along both the lateral and axial directions of the HIFU transducer to simulate respiration-induced motions during HIFU treatment, and the noise reduction level was investigated. In this experiment, the speed of movement was increased from 10 to 40 mm/s to simulate the actual movement of the tissue during HIFU exposure, with the intensity and driving frequency of HIFU set to 1.0–5.0 kW/cm^2^ and 1.67 MHz, respectively. To investigate the failure conditions of the proposed algorithm, the proposed method was applied with the HIFU focus located at the boundary between the phantom and water to easily cause cavitation bubbles. The intensity of HIFU was set to 10 kW/cm^2^.

**Results:**

Almost all HIFU noise was constantly able to be eliminated using the proposed method when the phantom was moved along the lateral and axial directions during HIFU exposure. The noise reduction level (PRL in this study) at an intensity of 1.0, 3.0, and 5.0 kW/cm^2^ was in the range of 28–32, 38–40, and 42–45 dB, respectively. On the other hand, HIFU noise was not basically eliminated during HIFU exposure after applying the proposed method in the case of cavitation generation at the HIFU focus.

**Conclusions:**

The proposed method can be applicable even if homogeneous tissues or organs move axially or laterally to the direction of HIFU exposure because of breathing. A condition under which the proposed algorithm failed was when instantaneous tissue changes such as cavitation bubble generation occurred in the tissue, at which time the reflected continuous wave response became less steady.

## Introduction

High-intensity focused ultrasound (HIFU) treatment is one of the less invasive surgeries for treating cancer, where ultrasound is generated and focused outside the body and induces a temperature rise at the target tissue [1–5]. In HIFU treatment, it is important to monitor the conditions of the target tissue before, during, and after HIFU exposure. There are mainly two modalities for monitoring HIFU treatment. One is magnetic resonance imaging (MRI) [6, 7] and the other is ultrasound imaging [8–10]. MRI can detect the temperature rise during HIFU treatment, but it performs with lower temporal resolution than ultrasound imaging. We have been studying a method to ultrasonically detect the treated area resulting from HIFU exposure because of the advantages of ultrasound in terms of its relatively higher temporal resolution, portability, and inexpensiveness.

In ultrasonic imaging for HIFU treatment, it is difficult to detect tissue changes on the order of milliseconds during HIFU exposure because therapeutic ultrasound interferes with the tissue (diagnostic) signals. In a previous study [11, 12], a noise elimination method using the continuous wave (CW) response of HIFU was proposed and applied to static cases where the target tissue sample was not moved. Another suggested noise reduction method for HIFU treatment using the notch filter or pulse inversion exposure was also applied to cases without respiration, which induced repetitive movement of the body and organs such as liver, kidney, and pancreas in actual surgery. Therefore, it is important to apply the proposed method to cases with respiration to demonstrate its clinical utility.

Bussels et al. [13] observed that the movement induced by respiration in the case of the liver, kidney, and pancreas was 24.4 ± 16.4 mm, 23.7 ± 15.9 mm, and 16.9 ± 6.7 mm, respectively, using MRI, and the maximum velocity of movement for the organs could be roughly estimated to be less than about 25 mm/s using their reported data. Balter et al. [14] also reported that the movement of the kidney was almost the same value using computed tomography (CT). The heart wall movement was also evaluated using Doppler imaging from cardiac tissue, and the velocity of ventricular posterior wall excursion was 20 ± 6 mm/s [15].

In the present study, an experimental setup to simulate repetitive movement of tissue induced by respiration was constructed, and the proposed method was applied to HIFU exposure experiments to evaluate the feasibility of this method for actual HIFU treatment. In actual treatment, respiration induces movements of the different organs anterior–posteriorly (axially) and laterally to the direction of HIFU exposure. Therefore, the clinical utility of this method for tissue movement in two directions (axial and lateral) was investigated and discussed in this study. In addition to that, the failure conditions of the proposed algorithm were also investigated.

## Materials and methods

### Tissue-mimicking phantom

In this study, polyurethane material (Exseal Corporation, Japan) was used to make the tissue-mimicking phantoms. Glass beads with a diameter of 100 µm were mixed with the phantom as inclusions to have tissue-like scatterers in the phantom. The concentration of glass beads was 10%. Table 1 shows the properties of the phantom. Speed of sound and acoustic attenuation of the phantom were 1420 m/s and 1.49 dB/cm/MHz, respectively. The size of the phantom was 50 × 50 × 50 mm. The polyurethane material has thermal tolerance and is thought to be suitable for HIFU exposure experiments, although the acoustic attenuation of polyurethane is higher than that of human tissue.

**Table 1:** Tissue-mimicking phantom material properties

Density (kh/m^3^)	1060
Speed of sound (m/s)	1420
Attenuation coefficient (Np/m)	17

### Experimental setup

**Fig. 1:**
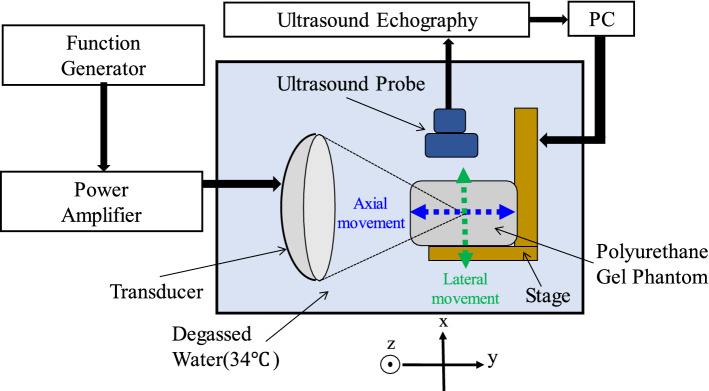
Schematic of experimental setup

**Fig. 2:**
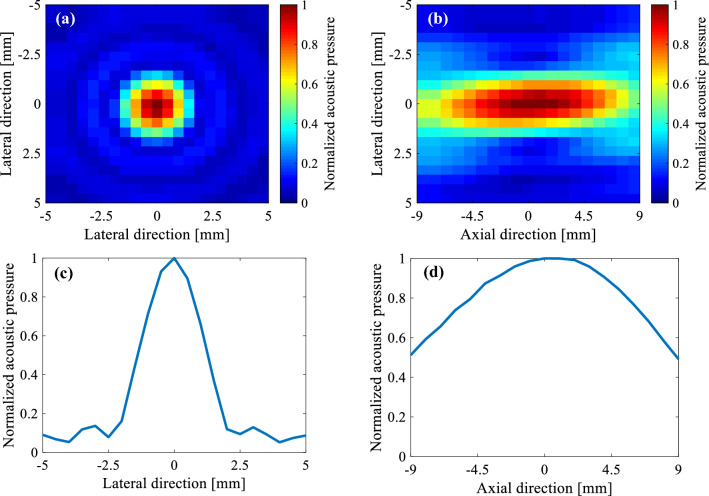
2-D HIFU focal pressure profiles in both **a** the transverse and **b** axial-lateral planes, and 1-D beam profiles along both **c** the lateral and **d** axial directions measured by needle hydrophone

Figure 1 shows a schematic of the experimental setup. HIFU was generated by a single-element concave transducer. The focal length, aperture, and driving frequency were 46 mm, 46 mm, and 1.67 MHz, respectively. The HIFU focus was set to be located in the phantom 20 mm from the surface. The driving signal (sinusoidal wave) was generated by a multifunction generator (WF1974; NF Corp., Japan) and amplified by an RF amplifier (2100L; E&I, Ltd., U.S.A). Figure 2 shows 2-D HIFU focal pressure profiles in both (a) the transverse and (b) axial-lateral planes, and 1-D beam profiles along both (c) the lateral and (d) axial directions measured by a needle hydrophone (NH0200; Precision Acoustics, U.K.). In this measurement, the spatial step size and spatial-peak temporal-peak intensity (I_SPTP_) of HIFU were 0.5 mm and 1 W/cm^2^, respectively. The polyurethane gel phantom was placed on a stage (KYL06050; SURUGA SEIKI Co., Ltd,, Japan), which was controlled by a PC. Ultrasonic RF signals were acquired by an ultrasound echography system (Vantage64; Verasonics Inc., USA) with a phased array probe (P4-2v; Verasonics Inc., USA). In this study, B-mode images were produced by applying plane wave transmission to detect relatively rapid changes such as cavitation bubble generation. The driving frequency of the probe was set at 2.5 MHz, and it was mounted above the tissue mimicking phantom. A water tank was filled with degassed water and kept at about 36℃ with a dissolved oxygen (DO) content of 30%.

### Experimental conditions and noise reduction method

#### Movement experiments

The phantom was moved along the lateral (*x*-axis in Fig. 1) and axial (*y*-axis in Fig. 1) directions of the HIFU transducer using the 2-D(XY) stage, which was controlled by a PC to simulate respiratory-induced movement. The start point of the movement was set to the HIFU focus, and the phantom was moved from − 10 mm to 10 mm along the lateral and axial directions around the HIFU focus. The velocity of the stage was increased to up to 40 mm/s in increments of 10 mm/s. These parameters are reasonable velocities for simulating respiratory-induced movement, as shown in previous articles [13–18]. In this experiment, the HIFU focus was set so that it was constantly located inside the phantom while the phantom was moving on the assumption of treatment in homogeneous tissue. The I_SPTP_ of HIFU was set at 1.0, 3.0, and 5.0 kW/cm^2^ so as not to cause cavitation bubbles during HIFU exposure. These intensities were estimated from the focal pressure at 1 W/cm^2^ in the measurement of beam profiles in 2.2. This estimation assumed a quadratic relation between the amplifier output voltage and the intensity.

#### Fixed phantom experiments

**Fig. 3:**
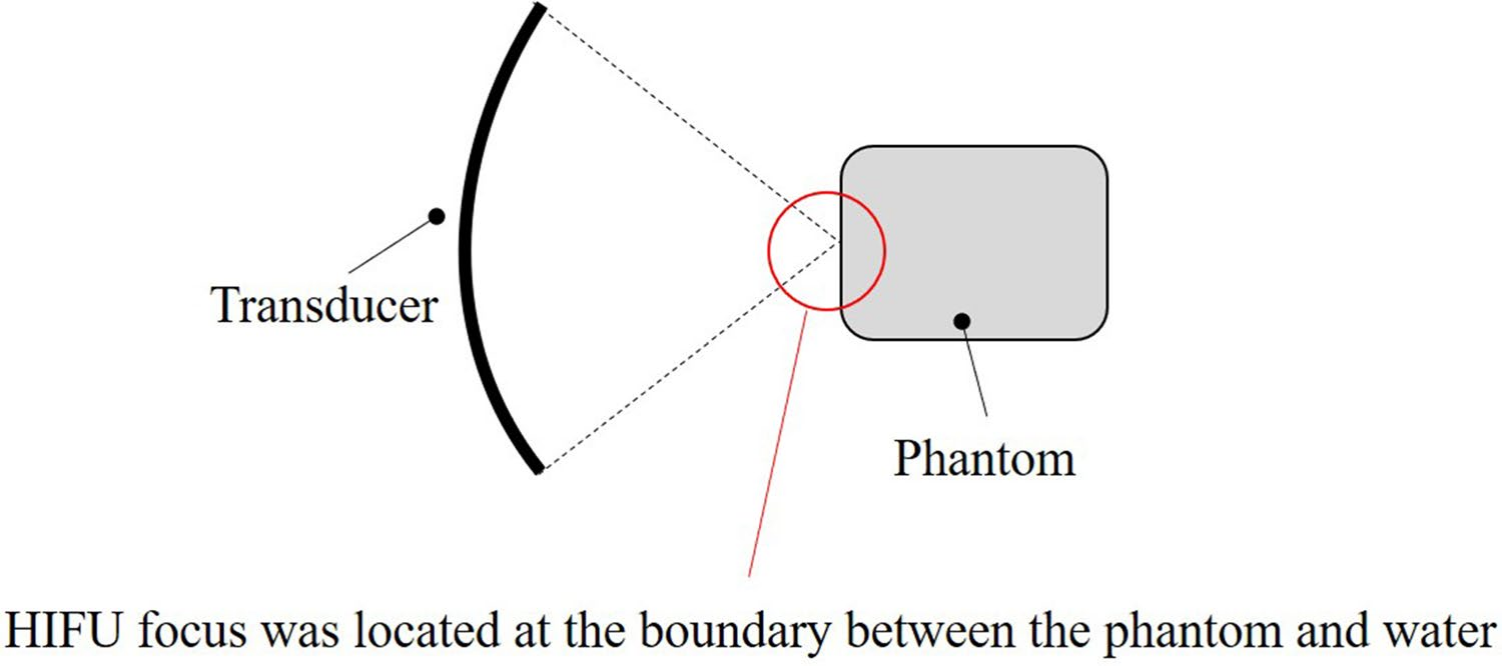
Experimental setup where the HIFU focus was located at the boundary between the phantom and water when HIFU was performed while the phantom was fixed (not moved)

To investigate fail conditions for the proposed algorithm, the HIFU focus was located at the boundary between the phantom and water, and HIFU was performed while the phantom was fixed (not moved), as shown in Fig. 3. This condition was set based on the assumption that rapid change in acoustic impedance (cavitation bubble generation) constantly occurs around the focal region during HIFU exposure. It is said that cavitation bubbles can easily occur at the boundary between two different mediums (acoustic impedance) in comparison to a homogeneous single tissue according to the literature [5, 19]. The intensity of HIFU was set to 10 kW/cm^2^ in this experiment.

#### Noise reduction method

In this study, the same algorithm as that in the preliminary study [11] was applied to the HIFU exposure experiments to eliminate HIFU noise in more practical cases in which the target phantom was moved. There are two types of received RF signals during ultrasound-guided HIFU exposure. One is the response to HIFU, which is the received CW component after HIFU signals are traveling through a medium such as water and tissue. The other is the pulse response to the imaging exposure, which is the received pulse component after the imaging pulse is reflected from the medium.

At a certain time after HIFU is initiated, the response to HIFU reaches the steady state, which is periodically repeated at a fundamental frequency of 1.67 MHz. All RF signals received in the time range corresponding to the water region are only the CW response to HIFU because there are no reflectors such as tissues. Therefore, the CW response to HIFU can be estimated from a portion of the RF signal with no pulse response to imaging exposure. The estimated CW response to HIFU was subtracted from the received RF signal to eliminate the CW response, while the pulse response to the imaging exposure remained [11].

**Fig. 4:**
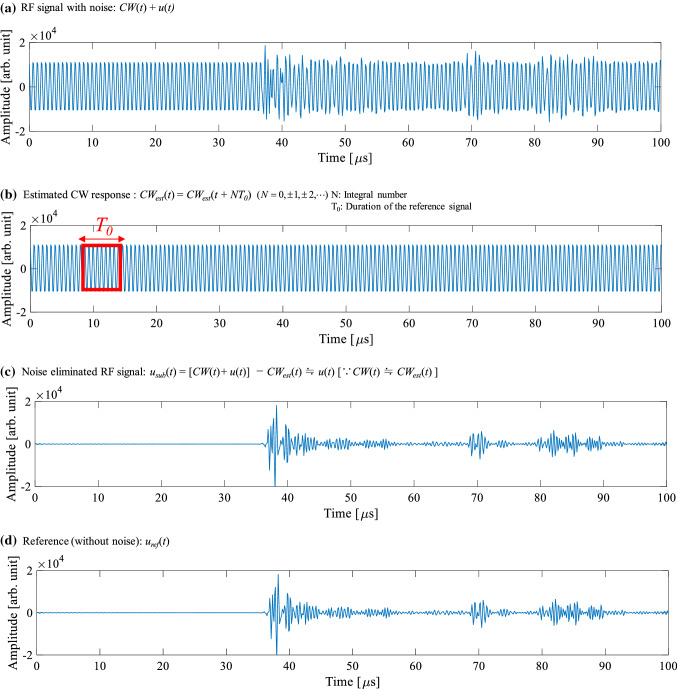
Diagram of noise elimination algorithm (RF signal received in a channel of the probe)

Figure 4 shows a diagram of the noise elimination algorithm using a RF signal received in a channel of the probe. Figure 4a shows a RF signal with noise received by the probe including both CW (*CW*(*t*)) and pulse response (*u*(*t*)). The entire duration of the received RF signal in a channel was about 100 µs in this experiment. Five periods of the fundamental component (about 4.5 µs) of the CW response in the water region (water balloon in the actual surgery) were periodically repeated in the time direction to estimate the CW response, as shown in Fig. 4b. The estimated CW response (*CW*_est_(*t*)) was subtracted from the RF signal with noise (*CW*(*t*) + *u*(*t)*) to eliminate only the CW response (*CW*(*t*)).

Figure 4c is the noise-eliminated RF signal (*u*_sub_(*t*)) after subtracting the estimated CW response (Fig. 4b) from the RF signal with noise (Fig. 4a). Figure 4d is only the pulse response without the CW response (noise). The RF signal shown in Fig. 4d is referred to as ‘Reference’ in this study.

When *CW*(*t*) is distorted and is in a non-steady state due to tissue movement within the entire duration of the received RF signal (about 100 µs), *CW*_est_(*t*) deviates from the *CW*(*t*) and HIFU noise remains.

#### HIFU sequence and data acquisition

**Fig. 5:**
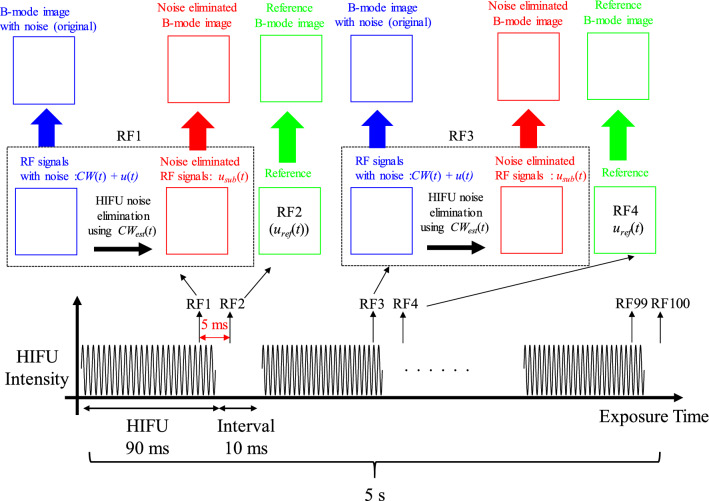
Sequence of HIFU exposure and RF signal acquisition

Figure 5 shows the sequence of HIFU exposure and RF signal acquisition. HIFU exposure was synchronized with the ultrasound echography system to set HIFU exposure duration with an intermission period to acquire RF signals both with and without HIFU noise (the CW response). The HIFU exposure duration and the following intermission period were 90 ms and 10 ms, respectively, and this sequence was repeated 50 times (5 s in total). The HIFU noise elimination algorithm was applied to the RF signals with noise (RF1, RF3,…RF99), which corresponds to *CW*(*t*) + *u*(*t)* in Fig. 4a, and noise-eliminated RF signals (*u*_sub_(*t*) in Fig. 4c) were acquired. RF signals (RF2, RF4,…RF100) were also acquired during the HIFU intermission period to evaluate the effectiveness of the noise elimination algorithm when tissue was moved. RF signals acquired during the HIFU intermission period were ‘References (*u*_ref_(*t*))’ in Fig. 4d. The acquisition interval between RF signals with and without HIFU noise was 5 ms. After acquiring and processing the RF signals, three types of B-mode images (original, noise-eliminated, and reference) at each time during HIFU exposure were generated, as shown in Fig. 5.

**Fig. 6:**
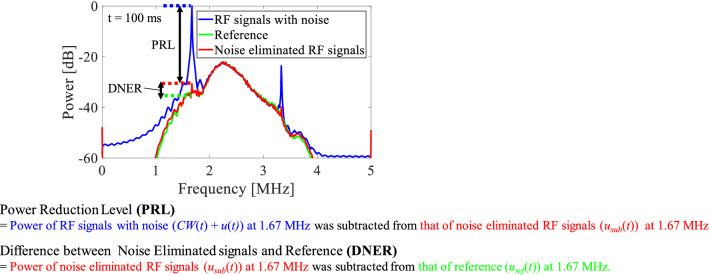
Channel-averaged frequency spectrum of original, noise-eliminated, and reference RF signals 100 ms after start of HIFU exposure, and the definition of two factors to evaluate the noise reduction level in this experiment

In this study, two factors were introduced using RF signals (channel data) before forming the B-mode images. Fast Fourier transform (FFT) was applied to the 64-channel RF signals at each time during HIFU exposure, and the frequency spectrum was calculated and averaged over all channels. Figure 6 shows the channel-averaged frequency spectrum of RF signals with noise (*CW*(*t*) + *u*(*t)*), noise-eliminated RF signals (*u*_sub_(*t*)), and reference RF signals (*u*_ref_(*t*)) received at 100 ms after start of HIFU exposure (RF1 and RF2 in Fig. 5).

The first factor, which is referred to as “power reduction level (PRL),” is the subtraction of channel-averaged RF signal power at the fundamental component of HIFU (1.67 MHz) between RF signals with noise (*CW*(*t*) + *u*(*t)*) and noise-eliminated RF signals (*u*_ref_(*t*)). PRL indicates how much HIFU noise was eliminated by applying the proposed method to the RF signals with noise. It is thought that PRL becomes larger when the reflected HIFU noise is increased. The second factor, which is referred to as “difference between noise-eliminated signals and reference (DNER),” is the subtraction of averaged RF signal power at the fundamental component of HIFU between the noise-eliminated (*u*_sub_(*t*)) and reference (*u*_ref_(*t*)) RF signals acquired during the HIFU intermission period. DNER indicates the similarity between B-mode images after noise elimination and without HIFU exposure (reference) because the acquisition interval between RF signals with and without HIFU noise was 5 ms, and it is thought that there are few phantom changes within such a short time according to the literature [21]. DNER becomes lower when HIFU noise is eliminated effectively using the proposed method, i.e., DNER should serve as an indicator for evaluating the robustness of the proposed method in the case of tissue movement. PRL and DNER were calculated and averaged for the entire HIFU exposure time (50 times), and the HIFU exposure experiment was repeated 15 times in total (*N* = 15).

## Results

### Movement experiments

**Fig. 7:**
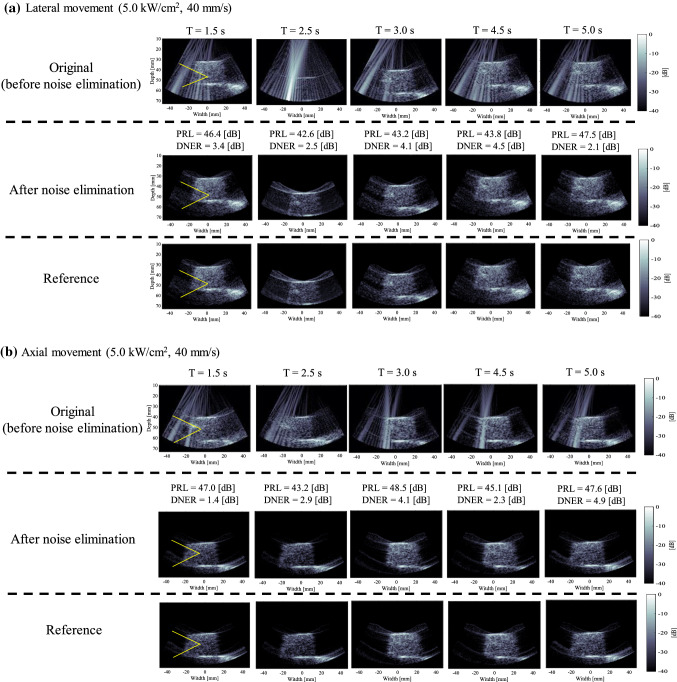
An example of time series of original, noise-eliminated, and reference B-mode images at an intensity of 5.0 kW/cm^2^ when the phantom was moved along the **a** lateral or **b** axial direction at a velocity of 40 mm/s

Figure 7 shows an example of time series of original, noise-eliminated, and reference B-mode images at an intensity of 5.0 kW/cm^2^ when the phantom was moved along the (a) lateral or (b) axial direction at a velocity of 40 mm/s. PRL and DNER at each time during HIFU exposure are also shown in Fig. 7. It was visually confirmed that HIFU noise was constantly reduced using the proposed method during HIFU exposure, as shown in Fig. 7. Cavitation bubbles were not observed in all cases.

**Fig. 8:**
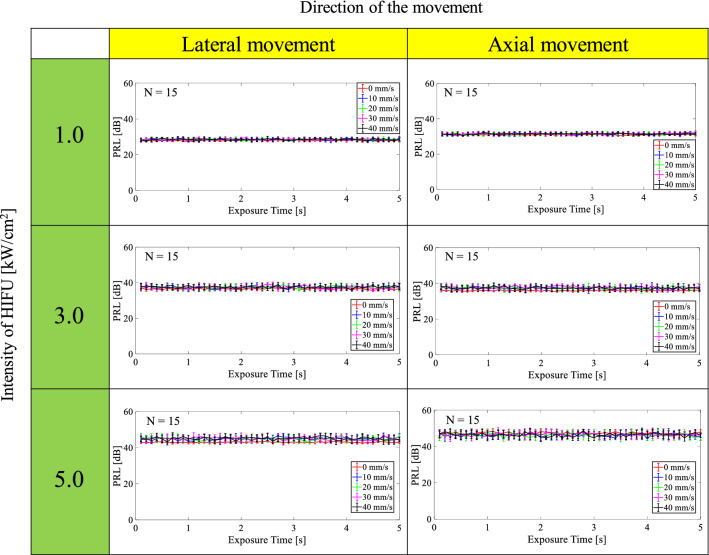
Time series of PRL at each intensity of HIFU (1.0, 3.0, and 5.0 kW/cm^2^) with and without movement along the lateral or axial direction

**Fig. 9:**
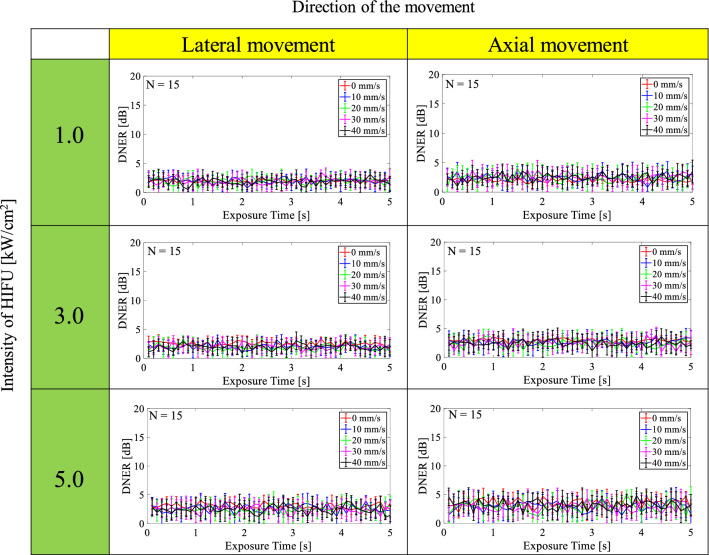
Time series of DNER at each intensity of HIFU (1.0, 3.0, and 5.0 kW/cm^2^) with and without movement along the lateral or axial direction

Figures 8 and 9 show time series of PRL and DNER at each intensity of HIFU (1.0, 3.0, and 5.0 kW/cm^2^) with and without movement along the lateral or axial direction. As shown in Fig. 8, PRL was relatively constant in time-direction regardless of the direction of movement and intensity. PRL at an intensity of 1.0, 3.0, and 5.0 kW/cm^2^ was in the range of 28–32, 38–40, and 42–45 dB, respectively. PRL became larger when the intensity was increased. The standard deviation of PRL in the case of no motion was relatively small compared with that in the case of motion, but median PRL was almost the same at each intensity. As shown in Fig. 9, DNER was also relatively constant in time-direction like PRL, and DNER was less than 5 dB in all cases. Figure 9 implies that almost all HIFU noise was constantly able to be eliminated using the proposed method when tissue was moved along the lateral and axial direction during HIFU exposure because DNER shows similarity between the noise-eliminated and reference RF signals (B-mode images).

### Fixed phantom experiments

**Fig. 10:**
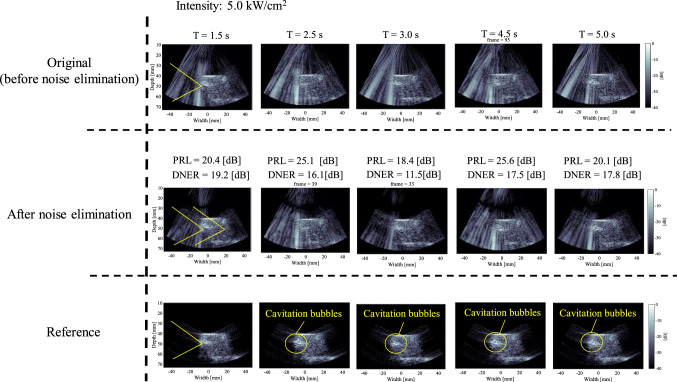
An example of time series of original, noise-eliminated, and reference B-mode images at an intensity of 5.0 kW/cm^2^ when the phantom was fixed and the HIFU focus was located at the boundary between the phantom and water

Figure 10 shows an example of time series of original, noise-eliminated, and reference B-mode images at an intensity of 10 kW/cm^2^ when the phantom was fixed and the HIFU focus was located at the boundary between the phantom and water. As shown in Fig. 10, cavitation bubbles were constantly observed at the HIFU focus during HIFU exposure. PRL and DNER at each time during HIFU exposure are also shown in Fig. 10. As shown in Fig. 10, HIFU noise was not eliminated completely and still remained in the B-mode images after the noise elimination processing.

**Fig. 11:**
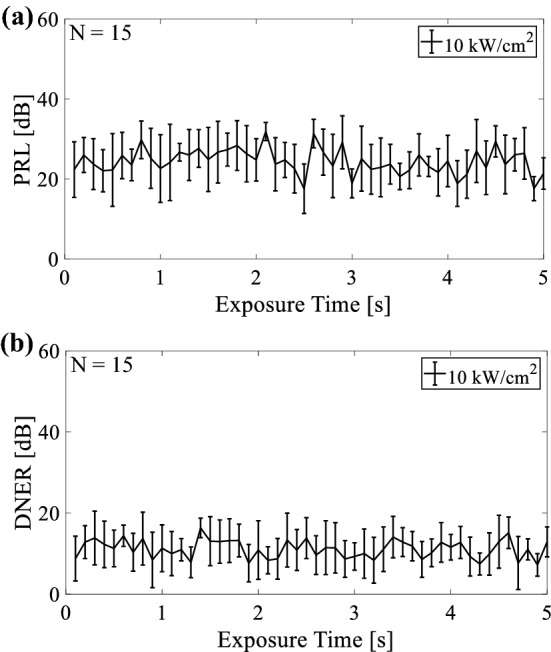
Time series of **a** PRL and **b** DNER at an intensity of 10 kW/cm^2^ when the phantom was fixed and the HIFU focus was located at the boundary between the phantom and water

Figure 11 shows the time series of (a) PRL and (b) DNER at an intensity of 10 kW/cm^2^ when the phantom was fixed and the HIFU focus was located at the boundary between the phantom and water. PRL and DNER changed markedly during HIFU exposure, as shown in Fig. 11. PRL and DNER were in the range of 13–35 dB and 2–20 dB, respectively. These results show that HIFU noise was not basically eliminated during HIFU exposure, and the difference between the noise-eliminated and reference RF signals (B-mode images) became large. As shown in Fig. 11b, DNER sometimes dropped below 5 dB, which means that HIFU noise was eliminated effectively despite the presence of cavitation bubbles at the HIFU focus.

## Discussion

### Movement experiments

As shown in Figs. 8 and 9, PRL and DNER were relatively constant in time-direction regardless of the direction of the movement and intensity. PRL became larger as the intensity of HIFU was increased because the reflected CW response increased with the intensity of HIFU. DNER, which is an indicator of similarity between noise-eliminated and reference B-mode images, was relatively small (less than 5 dB) in all cases. These results imply that almost all HIFU noise should constantly be eliminated using the proposed method even if homogeneous tissues or organs move axially or laterally to the direction of HIFU exposure because of breathing.

It is thought that the proposed algorithm fails when the CW response (*CW*(*t*)) becomes unsteady due to tissue movement within the entire duration of the RF signal (about 100 µs in this experiment) and the estimated CW response (*CW*_est_(*t*)) deviates from the *CW*(*t*).

In this study, the velocity of the movement was in the range of 10–40 mm/s, and the displacement of the phantom within the entire duration of the RF signal (100 µs) was 1–4 µm. It could be said that such a small displacement of a homogeneous phantom has no effect on the proposed method. The maximum velocity of the movement (40 mm/s) in this experiment was much higher than that of respiration-induced or heart wall movement, as described in the introduction, and so the proposed method has the robustness to withstand tissue movement due to breathing during actual HIFU treatment.

### Fixed phantom experiments

In HIFU treatment, thermal coagulation is induced by HIFU, and RF signals (B-mode images) change over time. The time-scale of RF signal change due to thermal coagulation is said to be 500 ms − 1 s [8, 9, 20], which is relatively longer than the entire duration of the RF signal (100 µs) to estimate the CW response. Therefore, it should be said that the RF signal change due to thermal coagulation has no effect on the estimation of the CW response within about 100 µs. However, the reflected RF signals (CW response) could be changed within about 100 µs when there is an instantaneous change in acoustic impedance such as cavitation and boiling during HIFU treatment.

In this study, the HIFU focus was located at the boundary between the phantom and water to simulate the condition that there is an instantaneous change in acoustic impedance induced by cavitation bubbles around the HIFU focus. As shown in Figs. 10, 11, HIFU noise was not eliminated and DNER was basically more than 10 dB, although the noise elimination processing was sometimes successful. This is thought to be because the received CW response within 100 µs was distorted by the sudden generation or violent oscillation of the bubbles and became unsteady.

**Fig. 12:**
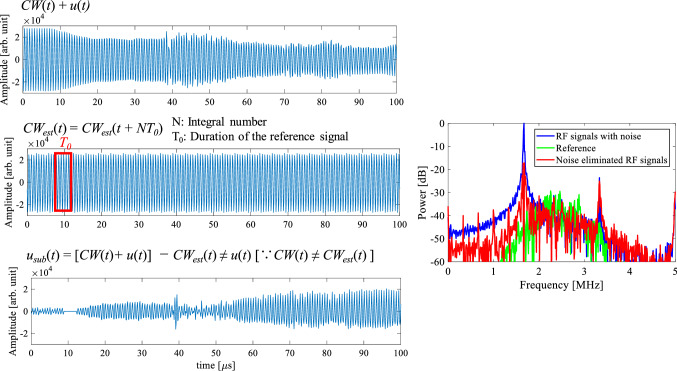
Examples of received RF signals in a channel and the frequency spectrum of original, noise-eliminated, and reference RF signals on the condition that the proposed algorithm failed

Figure 12 shows examples of received RF signals in a channel and the frequency spectrum of original, noise-eliminated, and reference RF signals on the condition that the proposed algorithm failed. As shown in Fig. 12, the estimated CW response deviated from the original CW response and HIFU noise remains.

In this experiment, the noise elimination processing was sometimes successful, which means that the CW response was not distorted despite cavitation bubbles around the HIFU focus. It is difficult to determine the reason for that in this study because the cavitation process under HIFU exposure is transient and very complicated. Further investigation using numerical simulation is needed to clarify the relationship between the bubble conditions under HIFU exposure and the reflected CW response.

Based on these results, it can be said that the proposed method could fail when there is an instantaneous change in acoustic impedance such as cavitation and boiling in tissue during actual HIFU treatment. To make the proposed method work well, surgeons should perform HIFU treatment by selecting the appropriate HIFU propagation path, where the mediums are relatively homogeneous and there are few strong ultrasound reflectors such as bones to cause cavitation, although sudden generation of cavitation and boiling in the treated region (HIFU focus) may be inevitable.

### PRL and DNER

DNER, which indicates the similarity between B-mode images after noise elimination and without HIFU exposure (reference), was introduced to evaluate the effectiveness of the proposed method in the experiments in this study. DNER should be 0 dB if HIFU noise is completely eliminated. However, DNER was actually in the range of 1–5 dB, which means that there were a few fundamental components of HIFU after applying the proposed method. Based on these results, it has to be thought that the estimated CW response very slightly deviated from the original CW response within 100 µs. However, it can be said that the difference between the noise-eliminated and reference B-mode images cannot be discriminated with the naked eye as long as DNER is less than 5 dB, as shown in Fig. 7, and the method can be applicable in actual surgery.

In actual HIFU treatment, the proposed method is applied to RF signals with noise during HIFU exposure without acquiring reference B-mode images, and PRL is a parameter that can be acquired during HIFU exposure.

It is difficult to confirm with the naked eye whether therapeutic ultrasound (HIFU) has been appropriately emitted or not based on the noise-eliminated B-mode images when the proposed method is working well during HIFU exposure. It can be confirmed that the target tissue has been exposed to HIFU by monitoring the magnitude of PRL even when HIFU noise is eliminated in the B-mode images because the magnitude of CW response is reflected on PRL.

**Fig. 13:**
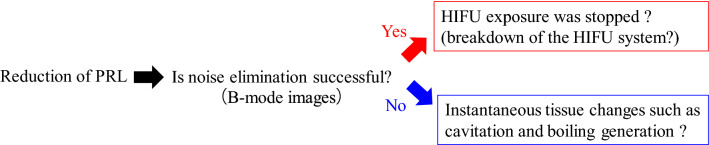
Concept of the usage of PRL

If PRL is suddenly reduced on the condition that there is no noise on B-mode images, HIFU may not be appropriately applied or stopped (breakdown of the HIFU system). On the other hand, rapid tissue changes (cavitation or boiling) may occur during HIFU exposure if PRL is suddenly reduced on the condition that there is uncancelled noise on the B-mode images. Therefore, PRL could be a parameter to support actual HIFU treatment. Figure 13 shows the concept of the usage of PRL.

## Conclusion

In this study, the developed noise reduction method was applied to cases where a tissue-mimicking phantom was moved to simulate respiration-induced movement to investigate the feasibility of this method for actual HIFU treatment. The proposed method can be applicable even if homogeneous tissues or organs move axially or laterally in the direction of HIFU exposure because of breathing. The fail conditions of the proposed algorithm were also investigated by setting the HIFU focus to the boundary between the phantom and water in this study. As a result, it was found that instantaneous tissue changes such as cavitation bubble generation occurred in the tissue, the reflected CW response became unsteady, and the proposed algorithm failed. Therefore, surgeons should perform HIFU treatment by selecting the appropriate HIFU propagation path where the mediums are relatively homogeneous and there are few strong ultrasound reflectors such as bones to cause cavitation in order to make the proposed method work well. The parameter PRL, which indicates how much HIFU noise is eliminated as a result of applying the proposed method, was introduced, and the utility of PRL in actual HIFU treatment was also investigated in this study.

The noise elimination processing was sometimes successful despite cavitation bubbles around the HIFU focus. To investigate the reason for that, further investigation is needed to clarify the relationship between the conditions of the bubbles under HIFU exposure and the reflected CW response.
